# Antimicrobial resistance genes harbored in invasive *Acinetobacter calcoaceticus-baumannii* complex isolated from Korean children during the pre-COVID-19 pandemic periods, 2015–2020

**DOI:** 10.3389/fcimb.2024.1410997

**Published:** 2024-07-04

**Authors:** Hyun Mi Kang, Kyung Ran Kim, Gahee Kim, Dong-gun Lee, Yae Jean Kim, Eun Hwa Choi, Jina Lee, Ki Wook Yun

**Affiliations:** ^1^ Department of Pediatrics, College of Medicine, Seoul National University, Seoul, Republic of Korea; ^2^ Department of Pediatrics, College of Medicine, The Catholic University of Korea, Seoul, Republic of Korea; ^3^ Vaccine Bio Research Institute, College of Medicine, The Catholic University of Korea, Seoul, Republic of Korea; ^4^ Department of Pediatrics, Sungkyunkwan University School of Medicine, Samsung Medical Center, Seoul, Republic of Korea; ^5^ Department of Pediatrics, Asan Medical Center, University of Ulsan College of Medicine, Seoul, Republic of Korea; ^6^ Department of Internal Medicine, College of Medicine, The Catholic University of Korea, Seoul, Republic of Korea; ^7^ Department of Pediatrics, Seoul National University Children’s Hospital, Seoul, Republic of Korea

**Keywords:** Acinetobacter baumannii, genotype, resistome, colistin resistance, children

## Abstract

**Background:**

*Acinetobacter baumannii* (AB) has emerged as one of the most challenging pathogens worldwide, causing invasive infections in the critically ill patients due to their ability to rapidly acquire resistance to antibiotics. This study aimed to analyze antibiotic resistance genes harbored in AB and non-*baumannii Acinetobacter calcoaceticus-baumannii* (NB-ACB) complex causing invasive diseases in Korean children.

**Methods:**

ACB complexes isolated from sterile body fluid of children in three referral hospitals were prospectively collected. Colistin susceptibility was additionally tested via broth microdilution. Whole genome sequencing was performed and antibiotic resistance genes were analyzed.

**Results:**

During January 2015 to December 2020, a total of 67 ACB complexes were isolated from sterile body fluid of children in three referral hospitals. The median age of the patients was 0.6 (interquartile range, 0.1–7.2) years old. Among all the isolates, 73.1% (n=49) were confirmed as AB and others as NB-ACB complex by whole genome sequencing. Among the AB isolates, only 22.4% susceptible to carbapenem. In particular, all clonal complex (CC) 92 AB (n=33) showed multi-drug resistance, whereas 31.3% in non-CC92 AB (n=16) (P<0.001). NB-ACB showed 100% susceptibility to all classes of antibiotics except 3rd generation cephalosporin (72.2%). The main mechanism of carbapenem resistance in AB was the *bla*_oxa23_ gene with ISAba1 insertion sequence upstream. Presence of *pmr* gene and/or mutation of *lpx*A/C gene were not correlated with the phenotype of colistin resistance of ACB. All AB and NB-ACB isolates carried the *abe* and *ade* multidrug efflux pumps.

**Conclusions:**

In conclusion, monitoring and research for resistome in ACB complex is needed to identify and manage drug-resistant AB, particularly CC92 AB carrying the *bla*_oxa23_ gene.

## 1 Introduction

Acinetobacter baumannii (AB) and its close relatives—*Acinetobacter calcoaceticus*, *Acinetobacter pittii*, *Acinetobacter nosocomialis*, *Acinetobacter seifertii*, and *Acinetobacter dijkshoorniae*—comprise the *Acinetobacter calcoaceticus-baumannii* (ACB) complex, which mainly causes hospital-acquired pneumonia and bacteremia (Towner, 2009; Chusri et al., 2014). The ACB complex is regarded as a group of phenotypically indistinguishable opportunistic pathogens. Among the ACB complexes, infections with AB are a major public health threat in health care settings worldwide (Doi et al., 2015). Non-*baumannii* ACB (NB-ACB) species also have more been reported as pathogens causing invasive infections than before (Chuang et al., 2011; Lee et al., 2011; Said et al., 2021).

In the recent decades, outbreaks caused by carbapenem resistant AB (CRAB) have been reported in many countries. Super-infection by carbapenem resistant AB and other gram negative organsims was observed in up to 20% of the patients hospitalized with coronavirus disease-2019 (COVID-19), and studies have shown that CRAB had independent association with 14-day mortality in COVID-19 respiratory sub-intensive care units (Casale et al., 2023; Iacovelli et al., 2023). The production of carbapenemases is the most common mechanism of carbapenem resistance, and so far, the most common carbapenemases found in AB are OXA-23 (Mugnier et al., 2010). In addition, CRAB isolates carrying NDM-1 have also been rapidly increasing worldwide (Chen et al., 2011; Pillonetto et al., 2014; Alcock et al., 2020). To make matters worse, there have been reports of a global emergence of resistant to colistin, which is essentially the last line of defense in treating extensively-drug resistant (XDR) AB (Cai et al., 2012).

In South Korea, expansion of ST191 and ST451 that belong to clonal complex (CC) 208 in adult patients from multiple centers have been observed. Clones that belong to CC208 have been shown to exhibit higher resistance to antimicrobials, therefore their expansion have been a global concern due to difficulties in treatment (Jun et al., 2021; Jun et al., 2023).

Currently most of the data on invasive AB infections are derived from adults, and data on the molecular epidemiology and antimicrobial resistance mechanism of AB and NB-ACB causing invasive infections in the pediatric population is very limited. Therefore, this study aimed to identify AB and NB-ACB genotypes causing invasive diseases in children and analyze their antibiotic resistome by using whole genome sequencing (WGS).

## 2 Methods

### 2.1 Subjects and isolates

All ACB complexes isolated from sterile body fluids in children who were admitted to three referral hospitals located in Seoul, South Korea, between 2015 and 2020 were collected prospectively. The specimens were collected from three hospitals during the patient’s routine clinical care and were transported to the microbiology unit of the laboratory in the corresponding hospitals for routine microbiological analysis. Identification of the ACB complex and antibiotic susceptibility test (AST) were performed using a VITEK2 automated system (BioMériux, Marcy l’Étoile, France). The results of AST were retrospectively collected and interpreted according to the Clinical and Laboratory Standards Institute (CLSI) guideline (CLSI, 2020). Multidrug resistance (MDR) was defined as resistance to at least one antimicrobial drug in three or more antimicrobial categories, and XDR was defined as non-susceptibility to all antimicrobial agents except in two or fewer antimicrobial categories. The isolates were stored at -80°C before the extraction of genomic DNA. Demographic and clinical information were collected from the medical chart review. The protocol for this study was approved by the institutional review board of Seoul National University Hospital (no. H-1812–080-995). The requirement for informed consent was waived due to the retrospective design.

### 2.2 Colistin susceptibility testing

Colistin susceptibility was additionally tested via broth microdilution (BMD) according to the CLSI-EUCAST guidelines. Minimum inhibitory concentrations (MICs) were determined using untreated MicroWell trays (Thermo Fisher Scientific, Inc., Waltham, MA, US) and 5×105 colony-forming units/mL of AB was inoculated. The plates were then incubated for 24 hours and read using the Sensititre Manual Viewer (Thermo Fisher Scientific, Inc., Waltham, MA, US). Colistin resistance was defined as MIC ≥4 μg/mL according to the CLSI criteria, and MIC <4 μg/mL was considered intermediate susceptible (CLSI, 2020).

### 2.3 Whole genome sequencing

Genomic DNA was prepared using the MasterPure™ Complete DNA Purification Kit (Lucigen, Middleton, WI, USA) from colonies grown on sheep blood agar plates according to the manufacturer’s instructions. The DNA was qualified using a NanoDropTM (ThermoFisher Scientific, Waltham, MA, USA) and was quantified using a 1X dsDNA HS Assay kit on a QubitTM 4 fluorometer (ThermoFisher Scientific). Equal amounts of DNA (~400 ng) from each isolate were used for library preparation using the Rapid Barcoding Sequencing Kit (SQK-RBK004) according to the manufacturer’s protocol (Oxford Nanopore Technologies, Oxford, UK). Pooled libraries of six isolates were run on a FLO-MIN106 flow cell with MinION next-generation sequencing (NGS) equipment and MinKNOW software v.4.1.22 (Oxford Nanopore Technologies) for 48 hours. Basecalling was performed on Guppy software v.4.0 (Oxford Nanopore Technologies), which was integrated into MinKNOW after a sequencing run had been completed. The base-called data were packed into FASTQ with a maximum of 4,000 reads per file. The FASTQ files were then uploaded to the EPI2ME cloud platform via EPI2ME Agent v3.1.3 (Oxford Nanopore Technologies) for the post-basecalling analyses.

### 2.4 Species identification

What’s in my pot (WIMP) is an EPI2ME workflow for the taxonomic classification of basecalled sequences/reads generated by Nanopore sequencing. The WIMP initially filters FASTQ files with a mean Q-score below a minimum threshold of 6. For reads above the quality threshold, the Centrifuge classification engine (RefSeq for bacteria) is executed to assign each read to a taxon in the National Center for Biotechnology Information taxonomy. The centrifuge classification results were then filtered and aggregated to calculate and report the counts of the reads at the species rank. Bacterial species were confirmed when the 1st-ranked species included >90% of the sequences that were tested.

### 2.5 Multilocus sequence typing

The seven multilocus sequence typing (MLST) gene (*cpn*60, *fus*A, *glt*A, *pyr*G, *rec*A, *rpl*B, and rpoB) sequences were automatically extracted from the draft WGS and were compared with the reference allele sequences in the database (https://pubmlst.org/organisms/acinetobacter-baumannii/) using CLC Genomics Workbench 7.0.4 (Qiagen). The sequence type (ST) was determined based on the Pasteur scheme of *Acinetobacter* species MLST. The BUST program in the PubMLST site (https://pubmlst.org/) was used to assign the STs to a CC, which was defined as STs differing from the confounding ST at one or two loci. The relationship between the STs was visualized with a phylogenetic tree on ST by the PhyloViz program (https://online.phyloviz.net/).

### 2.6 Antimicrobial resistance gene detection

Antimicrobial resistance (AMR) mapping application (ARMA pathway on EPI2ME program) analysis allows the detection of genes responsible for AMR. The AMR Comprehensive Antibiotic Resistance Database (CARD) component will align input reads with the minmap2 program against all of the reference sequences available in the CARD, which includes protein references, drug classes, and resistance ontologies that describe associated resistance mechanisms (Mugnier et al., 2010). Among the CARD resistance model types, the protein homolog model included most of the CARD genes (92.2%). Because of the relatively high error rate of Nanopore sequencing and the abundance in the AMR mechanism of the ACB complex, only protein homolog models were counted in this study. Additionally, presence and location of *ISAba* insertion sequences and quinolone resistance gene (*gyr*A and *par*C) mutations were manually inspected for all mapped and assembled sequences. For colistin resistance, the *pmr* and *lpx*A/C gene sequences were extracted and a comparative analysis was performed with the reference sequence, AB strain A1 (accession no. CP010781.1).

### 2.7 Statistical analysis

We performed statistical analyses using SPSS version 25.0 software for Windows (IBM SPSS, Chicago, IL). Rates and proportions were compared between the groups using the chi-square test or Fisher’s exact test where appropriate. A P value <0.05 was considered statistically significant.

## 3 Results

### 3.1 Study isolates

During January 2015 to December 2020, a total 67 ACB complexes (reported as AB from microbiology laboratory) were isolated from sterile body fluid cultures of pediatric patients from three tertiary university referral hospitals. The median age of the patients was 0.6 (interquartile range, 0.1–7.2) years old, 55.2% were male, all patients had underlying diseases, and the most common underlying diseases were preterm (28.4%) followed by non-malignant chronic disease (26.9%). The seven- and 30-day mortality were 23.9% and 29.8%, respectively (**Table 1**). Among all the isolates, 73.1% (n=49) were confirmed as AB, and 26.9% (n=18) were confirmed as NB-ACB complex by using Nanopore sequencing. Of the NB-ACB complex, *A. nosocomialis* was most commonly isolated (n=9), followed by *A. pittii* (n=7) and *A. seifertii* (n=2). Compared to patients infected with NB-ACB, patients with AB had a significantly higher seven-day (30.6% vs. 5.6%, P=0.033) and 30-day (38.8% vs. 5.6%, P=0.002) mortality rate (**Table 1**).

**Table 1:** Demographics of Korean children infected with *Acinetobacter calcoaceticus-baumannii* complex in this study.

	No. of cases (%)			*P*-value
	Total	AB	NB-ACB	
	(n=67)	(n=49)	(n=18)	
Sex, male	37 (55.2)	28 (57.1)	9 (50.0)	0.602
Median age (IQR), years	0.6 (0.1–7.2)	0.6 (0.0–7.7)	0.5 (0.1–3.8)	0.164
Underlying disease				0.019
Malignancy	14 (20.9)	13 (26.5)	1 (5.6)	
Congenital heart disease	13 (19.4)	10 (20.4)	3 (16.7)	
Preterm, ELBWI	19 (28.4)	15 (30.6)	4 (22.2)	
Solid organ transplant	3 (4.5)	3 (6.1)	–	
Non-malignant chronic disease	18 (26.9)	8 (16.3)	10 (55.6)	
Cultured specimen				0.241
Blood	62 (92.5)	45 (91.8)	17 (94.4)	
Pleural fluid	3 (4.5)	3 (6.1)	–	
Ascites fluid	1 (1.5)	–	1 (5.6)	
Cerebrospinal fluid	1 (1.5)	1 (2.0)	–	
Mortality				
7-day mortality	16 (23.9)	15 (30.6)	1 (5.6)	0.033
30-day mortality	20 (29.8)	19 (38.8)	1 (5.6)	0.002

AB, Acinetobacter baumannii; NB-ACB, Non-baumannii Acinetobacter calcoaceticus-baumannii; ELBWI, extremely low birth weight infant; IQR, interquartile range.

### 3.2 Genotype diversity

**Figure 1:**
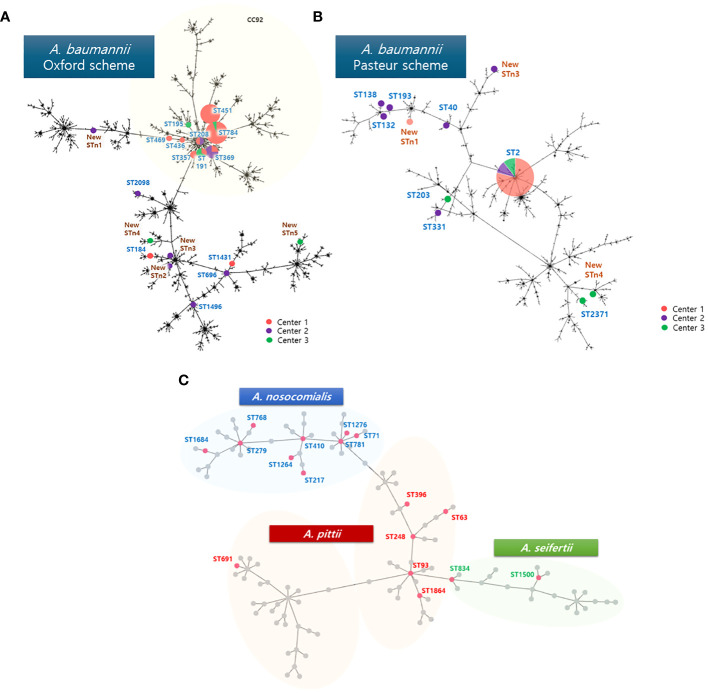
Phylogenetic tree with **(A)** sequence types of AB analyzed with Oxford scheme, **(B)** Pasteur scheme, and **(C)** non-AB ACB complex analyzed with Pasteur scheme in the MLST database. One new ST, STn2, could not be analyzed with the Pasteur scheme. The size of the circles represent the number isolated. Center 1, Seoul National University Children’s Hospital; Center 2, Asan medical center; Center 3, Samsung medical center; AB, *A baumannii*; CC, clonal complex; ST, sequence type.

Among AB, 33 (67.3%) isolates were assigned to CC92 by MLST using the Oxford scheme (**Figure 1A**). Although majority of the STs in the three hospitals differed, the following four STs were shared between hospitals; ST 191 (center 1, n=1; center B, n=2), ST 208 (center 1, n=1; center C, n=1), ST 369 (center 1, n=1; center 3, n=3), ST 784 (center 1, n=13; center 2, n=1).

Using the Pasteur scheme to analyze AB (**Figure 1B**), we found that the study isolates had less discriminatory power compared to the Oxford scheme. A total 8 STs were observed, with ST 2 shared by all three centers (center 1, n=30; center 2, n=4; center 3, n=4). Other STs included one isolate each: ST 40, ST 132, ST 138, ST 193, ST 203, ST 331, and ST 2,371.

All NB-ACB isolates had different STs except for two *A. pittii* isolates, which were both ST63. For the three NB-ACB species identified in the current study, ST phylogeny showed a clearly separated distribution and relationship (**Figure 1C**).

### 3.3 Comparison of antimicrobial susceptibility of the AB versus NB-ACB

The AB isolates showed an overall low susceptibility to all tested antibiotics, with only 22.4% (n=11/49) susceptible to carbapenem, 26.9% (n=7/26) to amikacin, and 38.8% (n=19/49) to trimethoprim-sulfamethoxazole. Furthermore, 28.6% (n=14/49) had resistance to colistin. An overall 77.6% (n=38/49) were MDR, and 71.4% (n=35/49) were XDR. Within the AB isolates, a significant difference in antimicrobial susceptibility was observed between CC92 and non-CC92. The percentage of MDR was 100% (n=33/33) in CC92 and 31.3% (n=5/16) in non-CC92 (P<0.001). NB-ACB showed 100% susceptibility to all classes of antibiotics except 3rd generation cephalosporin, where 5 isolates (2 A*. nosocomialis* and 3 A*. pittii*) showed intermediate susceptibility (MIC=16 mg/L). One *A. seifertii* isolate also showed resistance to colistin (MIC>4 mg/L) (**Table 2**).

**Table 2:** Antimicrobial susceptibility (%) of the *Acinetobacter calcoaceticus-baumannii* complexes.

	No. of cases/Total cases (%)											
	AMP/ SUL	PIP	PIP/ TAZ	CTX	CFP	MPM	AMK	CIP	TMP/ SMZ	COL	MDR	XDR
*A. baumannii* (n=49)	11/22 (50.0)	9/49 (18.4)	11/49 (22.4)	11/22 (50.0)	10/49 (20.4)	11/49 (22.4)	7/26 (26.9)	10/49 (20.4)	19/49 (38.8)	35/48 (72.9)	38/49 (77.6)	35/49 (71.4)
CC92 (n=33)	0/6 (0)	0/33 (0)	0/33 (0)	0/6 (0)	0/33 (0)	0/33 (0)	0/19 (0)	0/33 (0)	7/33 (21.2)	21/33 (63.6)	33/33 (100)	30/33 (90.9)
Non-CC92 (n=16)	11/16 (68.8)	9/16 (56.3)	11/16 (68.8)	11/16 (68.8)	10/16 (62.5)	11/16 (68.8)	7/7 (100)	10/16 (62.5)	12/16 (75.0)	14/16 (87.5)	5/16 (31.3)	5/16 (31.3)
Non-AB ACB (n=18)	18/18 (100)	18/18 (100)	18/18 (100)	13/18 (72.2)	18/18 (100)	18/18 (100)	18/18 (100)	18/18 (100)	18/18 (100)	17/18 (94.4)	0/18 (0)	0/18 (0)
* A. nosocomialis* (n=9)	9/9 (100)	9/9 (100)	9/9 (100)	7/9 (77.8)	9/9 (100)	9/9 (100)	9/9 (100)	9/9 (100)	9/9 (100)	9/9 (100)	0/9 (0)	0/9 (0)
* A. pittii* (n=7)	7/7 (100)	7/7 (100)	7/7 (100)	4/7 (57.1)	7/7 (100)	7/7 (100)	7/7 (100)	7/7 (100)	7/7 (100)	7/7 (100)	0/7 (0)	0/7 (0)
* A. seifertii* (n=2)	2/2 (100)	2/2 (100)	2/2 (100)	2/2 (100)	2/2 (100)	2/2 (100)	2/2 (100)	2/2 (100)	2/2 (100)	1/2 (50.0)	0/2 (0)	0/2 (0)

AMP/SBT, ampicillin-sulbactam; PIP, piperacillin; PIP/TAZ, piperacillin-tazobactam; CTX, cefotaxime/ceftriaxone; CFP, cefepime; MPM, meropenem; AMK, amikacin; CPR, ciprofloxacin; TMP/SMX, trimethoprim-sulfamethoxazole; MDR, multidrug resistance; XDR, extensive drug resistance; AB, Acinetobacter baumannii; ACB, Acinetobacter calcoaceticus-baumannii.

No of cases was based on isolates that were colistin intermediate (≤2 mg/L), according to the CLSI guideline8.

### 3.4 Antimicrobial resistance genes in AB and NB-ACB

#### 3.4.1 Beta-lactam

**Figure 2:**
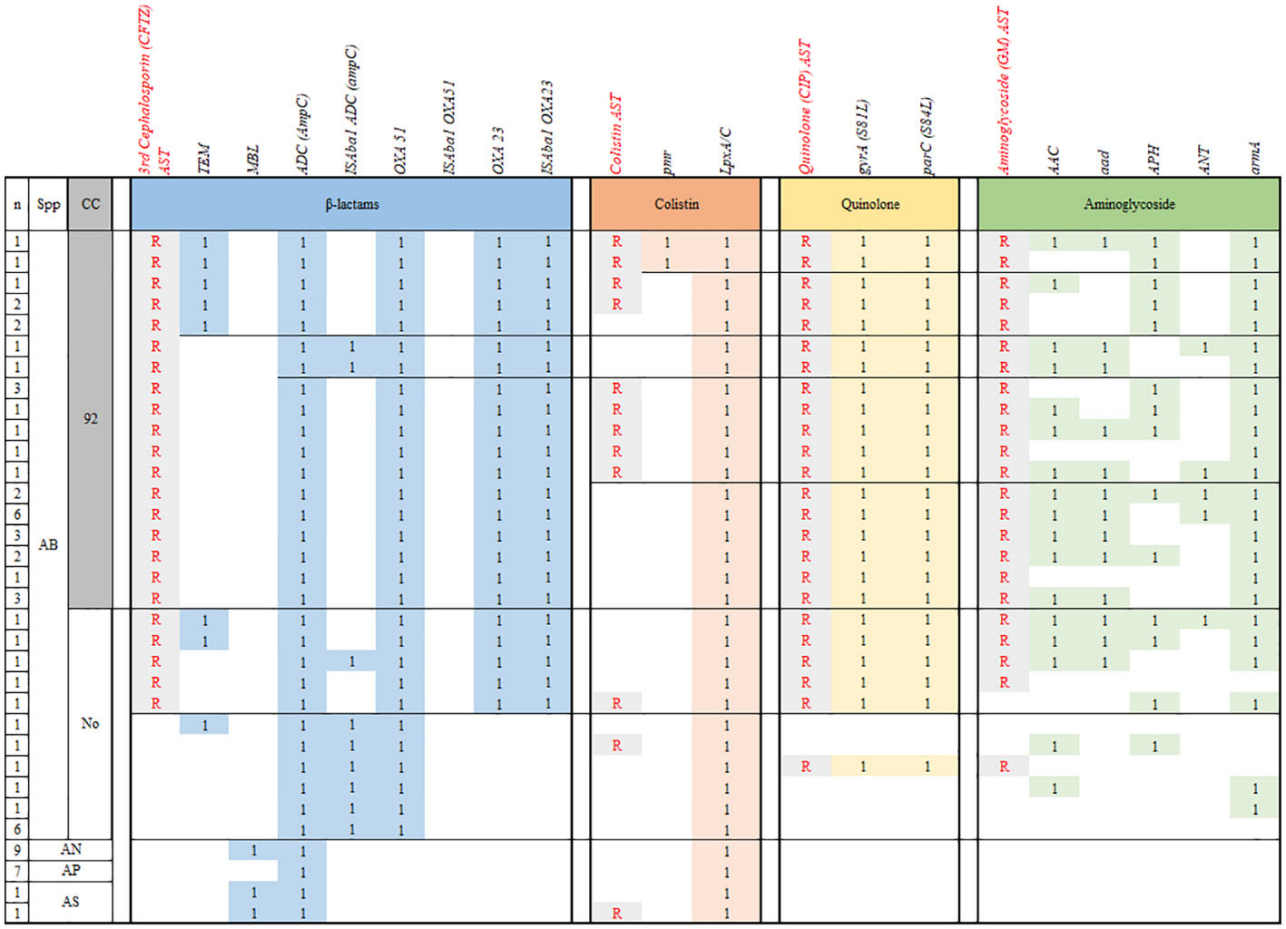
Comparison of the antibiotic resistome profiles of AB isolated from children with invasive infections. n, number; CC, clonal complex; CP, Chloramphenicol; CRAB, carbapenem-resistant *A. baumannii*; MDR, multidrug resistant; ML, macrolide; R, phenotypic resistance; SA, sulfonamide; SM, streptomycin; SMC, Samsung medical center; TC, tetracycline; 1, presence of resistance gene.

All AB isolates contained *bla*_ADC_, *bla*_OXA51_, and *bla*_OXA23_ genes, but the ISAba1 insertion sequence was detected at upstream of blaOXA23 gene in all CRAB and no carbapenem-susceptible (CS) AB. Three (9.1%) and none of CRAB carried *ISAba*1 upstream to *bla*_ADC_ and *bla*_OXA51_, respectively. All (n=11) CSAB, which included in non-CC92 AB, carried *ISAba1* upstream to *bla*_ADC_, but none of them showed resistance to 3rd-generation cephalosporin. Among 10 AB harboring *bla*_TEM_, all but one isolates also harbored *ISAba*1-*bla*_OXA23_, and thus were MDR as well as CRAB. The other AB isolate was non-CC92 and showed exclusive susceptibility to all antibiotics (**Figure 2**).

In NB-ACB isolates, all carried *bla*_ADC_ gene without upstream *ISAba*1. The metallo-β-lactamase (MBL) gene was detected in all of the *A. nosocomialis* and *A. sefertii* isolates, whereas the *bla*_OXA421_ gene was detected in all of the *A. pittii* isolates. None of *bla*_MBL_ MBL and *bla*_OXA421_ genes were accompanied with the upstream *ISAba*1 and none of NB-ACB isolates with these β-lactamases showed a resistance to β-lactam antibiotics including carbapenem (**Table 3**).

**Table 3:** Antimicrobial resistance genes detected by whole genome sequencing.

		AB, n (%)		NB-ACB, n (%)		
		CC92 (n=33)	Non-CC92 (n=16)	AN (n=9)	AP (n=7)	AS (n=2)
Beta-lactams	*TEM*	7 (21.2)	3 (18.8)	–	–	–
	*MBL*	–	–	9 (100)	–	2 (100)
	*ADC (ampC)*	33 (100)	16 (100)	9 (100)	7 (100)	2 (100)
	*ISAba1*	2 (6.1)	12 (75.0)	–	–	–
	*OXA51*	33 (100)	16 (100)	–	–	–
	*ISAba1*	–	–	–	–	–
	*OXA23*	33 (100)	5 (31.3)	–	–	–
	*ISAba1*	33 (100)	5 (31.3)	–	–	–
	*OXA421*	–	–	–	7 (100)	–
	*ISAba1*	–	–	–	–	–
Colistin	*pmr*	2 (6.1)	–	–	–	–
	*lpxA/C*	33 (100)	16 (100)	–	–	–
Quinolone	*gyrA*	33 (100)	16 (100)	9 (100)	7 (100)	2 (100)
	*parC*	33 (100)	16 (100)	9 (100)	7 (100)	2 (100)
	*patA*	1 (3.0)	–	–	–	–
	*emr*	3 (9.1)	–	–	–	–
Amino-glycoside	*aac*	23 (69.7)	5 (31.3)	–	–	–
	*aad*	21 (63.6)	3 (18.8)	–	–	–
	*ant*	10 (30.3)	3 (18.8)	–	–	–
	*aph*	16 (48.5)	4 (25.0)	–	–	–
	*armA*	33 (100)	6 (37.5)	–	–	–
Multi-drug efflux pump	*Abe*	33 (100)	33 (100)	9 (100)	7 (100)	2 (100)
	*Ade*	33 (100)	33 (100)	9 (100)	7 (100)	2 (100)
	*mexT*	33 (100)	33 (100)	9 (100)	7 (100)	2 (100)

AB, Acinetobacter baumannii; AN, A. nosocomialis; AS, A. seifertii; AP, A. pittii; NB-ACB, Non-baumannii Acinetobacter calcoaceticus-baumannii.

#### 3.4.2 Colistin

The *pmr* gene was detected in two of 14 colistin-resistant (Col-R) AB isolates and none of 25 colistin-susceptible (Col-S) AB and 18 NB-ACB isolates. Plasma-encoded *mcr* gene was not detected in any of the isolates. Meanwhile, all AB and NB-ACB isolates harbored both the *lpx*A and *lpx*C genes. Although several single nucleotide polymorphisms were observed in *lpx*A and *lpx*C genes from ACB isolates in this study, all were synonymous mutations (**Supplementary Figures 1, 2**). Any other AMR genes detected were significantly different in frequency between Col-R and Col-S ACB isolates (**Table 3**).

#### 3.4.3 Others

For fluoroquinolone resistance, the presence of both mutations in *gyr*A and *par*C causing antibiotic target alteration (S81L and S84L, respectively) was exclusively matched to phenotypical quinolone-resistance in AB (n=38, 77.6%; **Figure 2**). No NB-ACB isolate showed quinolone-resistance and mutations in *gyr*A and *par*C genes. A variety of resistance genes to aminoglycoside were carried in AB isolates: antibiotic inactivation genes (*aac*, *aad*, *APH*, *ANT*), antibiotic target alteration genes (*arm*A), and resistance-nodulation-cell division (RND) antibiotic efflux pumps (*sme*B and *amr*B). All AB isolates carried the abe and ade efflux pumps, regardless of resistance to aminoglycoside. The AMR genes most closely correlated with the aminoglycoside resistance were *aad* and *arm*A genes: 63.2% and 94.7% in aminoglycoside-resistant (n=38) and 0% and 27.3% in aminoglycoside-susceptible AB isolates (n=11), respectively. NB-ACB isolates were exclusively susceptible to aminoglycoside, and only *abe* and *ade* efflux genes were detected in all these isolates (**Table 3**).

## 4 Discussion

AB has emerged as a major global threat, especially due to its exceptional ability to acquire resistance genes to all classes of antibiotics currently available. This study aimed to identify circulating AB and NB-ACB genotypes causing invasive diseases in Korean children, analyze their antibiotic resistomes, and investigate the mechanism of carbapenem and colistin resistance. Compared to NB-ACB isolates, AB had a significantly higher mortality rate with an overall low susceptibility to antibiotics, with carbapenem and colistin resistances in 77.6% and 27.1% of the isolates, respectively. In particular, CC92 AB were exclusively CRAB and MDR, with the main resistance mechanism found to be OXA23 β-lactamase. Colistin resistance was not correlated with the presence of *pmr* gene and/or mutations in *lpx*A/C genes.

ACB complex is usually reported as AB from the laboratory using commercial bacterial identification system. However, correctly identifying ACB subspecies is important because of the poorer prognosis of AB than NB-ACB infections (Kuo et al., 2013; Xiao et al., 2017; da Silva et al., 2018). In Japan, between 2001 and 2014, among 155 invasive *Acinetobacter* species strains, 27.1% was *A. pittii* and 25.8% was AB, and imipenem nonsusceptibility was detected only in 4 strains (Kiyasu et al., 2020). In the previous study in Mexico, a total of 88 strains were identified as ACB complex by VITEK II during 2015–2017. Among them, AB accounted for 89.8%; *A. pittii*, 6.8%; and *A. nosocomialis*, 3.4%. In addition, 44.3% were MDR strains and, 11.4% were XDR (Mancilla-Rojano et al., 2020). In the study from Russia and Kazakhstan in 2016–2022, 234 *Acinetobacter* isolates were identified as NB-ACB isolates, which comprised 6.2% of *Acinetobacter* spp. Most NB-ACB isolates were susceptible to all antibiotics; however, sporadic isolates were resistant to carbapenems (Sheck et al., 2023). As shown above, the proportion of NB-ACB strain and its subspecies among *Acinetobacter* species and their AMR vary according to country, period, specimen type, and isolation method. Also, in the current study, among the 68 invasive isolates reported as AB, one-fourth was NB-ACB, which had much lower antimicrobial resistance and mortality than AB. Among the NB-ACB species, the most abundant were *A. nosocomialis* and *A. pittii* as in the previous studies (Mancilla-Rojano et al., 2020; Sheck et al., 2023).

AB emerged as a global pathogen due to the successful expansion of a few epidemic lineages producing acquired OXA-type carbapenemases. Particularly ST2 (Pasteur scheme), known as international clone (IC) II, the most common clone globally, are distributed in Europe, Asia, and Latin America (Levy-Blitchtein et al., 2018; Villalon et al., 2019). Genotype replacement with CC92 (Oxford scheme), which is the same strain to ST2 (Pasteur scheme), has been a global phenomenon, especially in China where CC92 CRAB harboring *bla*_OXA23_ has been increasing (Ruan et al., 2013; Chen et al., 2017). The spread of this drug-resistant pathogen should be closely monitored and managed, particularly in critical care. In a previous single center study in Korea, 27 AB were isolated from blood of patients in PICU, and MLST analysis showed that ST138 (Oxford scheme, CC92) was predominant (70%) (Kim et al., 2022). Also in the current study, majority of AB were ST2, which showed XDR characteristics, with compete resistance to 3rd/4th generation cephalosporin, piperacillin-tazobactam, amikacin, ciprofloxacin, and even carbapenem. Furthermore, 36.4% were resistant to colistin.

In our previous study, we identified carbapenem nonsusceptibility as a risk factor for mortality due to AB in children. An early administration of appropriate antibiotics should be enacted, especially in children with neutropenia (Choe et al., 2019). The *bla*_OXA-23_ gene is one of the most prevalent β-lactamase genes on the genome of CRAB and is distributed worldwide (Mugnier et al., 2010; Ruan et al., 2013). Among 86 AB isolates obtained during 2008–2015 in Central Illinois, 70.9% were nonsusceptible to carbapenems. Among the CRAB isolates, *bla*_OXA-23_ was the most frequently detected carbapenemase gene (52%) (Koirala et al., 2020). However, it is suggested that *ISAba1* is providing the promoter for *bla*_OXA-51_ and *bla*_OXA-23_ genes (Turton et al., 2006). In the current study, the presence of *bla*_OXA-23_ genes and *ISAba*1 upstream of *bla*_OXA-23_ were exactly matched to carbapenem resistance in AB. Any other β-lactamase or other AMR mechanism could be differentiated between CRAB and CSAB. We may conclude that the main mechanism of carbapenem resistance in AB isolated in Korean children during 2015–2020 was *ISAba*1-*bla*_OXA-23_. Because the detection of AB with *bla*_OXA-23_ and MDR phenotye is associated with increased mortality, prompt identification and management is extremely important in the clinical field. Similar results of high mortality associated with MDR AB carrying *bla*_OXA-23_ was observed globally in different STs, and the *bla*_OXA-23_ gene found to be present on a conjugative transferable plasmid indicated the advantage in transferability of the genetic element (Lopes et al., 2015; da Silva et al., 2018).

AmpC β-lactamase coded in *bla*_ADC_ gene could induce resistance to 3rd-generation cephalosporin of gram-negative bacteria during the use (Tamma et al., 2019). In the current study, all ACB complex isolates harbored *bla*_ADC_ gene, but they did not show resistance to 3rd-genderation cephalosporin unless they also had *ISAba*1-*bla*_OXA-23_, regardless of the presence of upstream *ISAba*1. Thus, we may conclude that the harboring of *bla*_ADC_ itself is not the requirement of AMR, and upstream *ISAba*1 is not sufficient to activate *bla*_ADC_ within AB.

Because colistin is usually the only remaining treatment available, the emergence of colistin-resistant CRAB is devastating. Colistin resistance due to the acquisition of plasmid-mediated mobile colistin-resistant (mcr) genes has been described in numerous Enterobacterales, especially *Klebsiella pneumonia*, however also found in AB. In *Pseudomonas aeruginosa*, mcr-5 has been identified, however, activation of histidine kinase (PmrB) or the response regulaor (PmrA) following an amino-acid substitution has been found to be a key mechanism for colistin resistance (Liu et al., 2016; Jeannot et al., 2021; Novović and Jovčić, 2023). To date only chromosomally encoded colistin resistance mechanisms have been reported in AB. It is suggested that mutations in the *pmr* and *lpx* genes confer colistin resistance via modification and complete loss of lipopolysaccharide, respectively (Adams et al., 2009; Moffatt et al., 2010; Nie et al., 2020). In the current study, two isolates were found to harbor the *pmr* gene. The significant mutations in *pmr*A gene related to colistin-resistance (D82G and S119T) were not found, but mutations in *pmr*B gene (G21V and V227A) were found in both isolates. Furthermore, *lpx*A and *lpx*C were found in all the isolates, but the change in the amino acid sequence of *lpx*A found in one isolate, which were unlikely to be associated with colistin resistance mechanisms in our isolates. This shows that there are other mechanisms that have not yet been uncovered, that are associated with colistin resistance.

The most common mechanism of resistance to fluoroquinolone in AB involves alterations in the genes that encode subunits of the quinolone targets DNA *gyr*A and *par*C (Park et al., 2011). Phenotypical quinolone resistance was perfectly matched to both *gyr*A and *par*C gene mutations in the current study. All quinolone resistant AB were CRAB, which harbored *ISAba*1-*bla*_OXA-23_. None of CSAB had either *gyr*A or *par*C gene mutations.

There are two main mechanisms underlying aminoglycoside resistance in AB: aminoglycoside-modifying enzymes, which were encoded by *aac*, *aad*, *ant*, and *aph* genes, and antibiotic target alteration by 16S rRNA methylases (encoded by *arm*A) (Bakour et al., 2014). In this study, genes encoding aminoglycoside-modifying enzymes were found in 30.3%~69.7% of CC92 AB, 18.8%~31.3% of non-CC92 AB, and none of NB-ACB. The *arm*A gene was also detected in 100%, 37.5%, and 0%, respectively. In a previous report from 2 hospitals in South Korea, invasive AB isolates from adults during 2004–2005 showed 85.2% of harboring *arm*A (Cho et al., 2009).

The resistance-nodulation-cell division (RND) pumps are the most prevalent efflux pumps in AB and one of the most important determinants of MDR. Typically, efflux pumps (especially those chromosomally encoded) are controlled by negative regulators. Mutations within the negative regulators which releases the ‘block’ on the efflux pump leads to increased expression of the pump causing antibiotics resistance (Fernando and Kumar, 2013; Davin-Regli et al., 2021; Kornelsen and Kumar, 2021). A high prevalence of this pump was observed in up to 97% of MDR AB isolates worldwide (Nowak et al., 2015). In the invasive AB strains from this study, the *ade* RND pumps were present in 100%. Furthermore, *abe*, which is a chromosomally encoded drug efflux pump of the small multidrug resistance (SMR) family, was also found in 100% of the isolates in this study. Although just the presence of these efflux pumps did not correlate with MDR in AB, it might show us the potential of AB to be MDR pathogen whenever they get the certain promotor.

Rapid detection of AB and main carbapenemase genes can allow prompt intervention in patients with invasive infections. By identifying whether the identified AB is MDR phenotype, early initiation of adequate antibiotics can improve patient outcome. Commercial molecular rapid testing are available and have already implemented in routine diagnostics in many centers (Dunbar et al., 2022). Improvements have been made in providing rapid phenotypic AST (Briggs et al., 2021), the benefits and limitations of these rapid platforms are currently under study and more studies are needed in critically-ill children.

There were several limitations of this study. First, although the three centers included were representative centers in South Korea with the highest number of beds for treating critically ill children in the pediatric intensive care unit, the resistance profiles of AB causing nosocomial infections may differ at each hospital throughout Korea. Second, the total number of cases included is limited, and because this was a multicenter study, we were unable to obtain all clinical data including information on treatment regimens and duration for all patients. However, this is the first study to investigate, via WGS, the antibiotic resistome profiles of AB dominant in strains isolated from critically ill children, and is deemed to be valuable for the monitoring and treatment of invasive AB infections.

To conclude, we found that CC92 AB have an exceptional ability to acquire a wide variety of antibiotic resistomes which enhance their AMR and survival fitness. Non-CC92 isolates also harbored a variety of resistance genes for which they were phenotypically susceptible to, depicting the necessity for continuous monitoring. Many efforts are needed to prevent their outbreak in critical patients, including methods such as decreasing overall antibiotic use and relieving selective pressure, strict contact precaution and hand hygiene of medical staff to prevent transmission, and environmental disinfection to decrease sources of possible infection. Carbapenem resistance in AB was likely induced by blaOXA-23 carbapenemases promoted by ISAba1. Although only one-third of the isolates were shown to be resistant to colistin, we found that all AB harbored mutations in enzymes that produce lipid A, meaning all AB have the potential to become resistant to colistin. Further studies are necessary to understand resistance mechanisms in order to combat the highly virulent XDR AB, and evaluate susceptibility to novel approved drugs such as cefiderocol and sulbactam/durlobactam.

## Data availability statement

Original datasets are available in a publicly accessible repository:The original contributions presented in the study are publicly available. This data can be found here: NCBI, PRJNA1127342.

## Ethics statement

The studies involving humans were approved by institutional review board of Seoul National University Hospital (no. H-1812-080-995). The studies were conducted in accordance with the local legislation and institutional requirements. Written informed consent for participation was not required from the participants or the participants’ legal guardians/next of kin in accordance with the national legislation and institutional requirements.

## Author contributions

HK: Conceptualization, Data curation, Formal Analysis, Investigation, Methodology, Validation, Writing – original draft, Writing – review & editing. KK: Data curation, Investigation, Methodology, Writing – review & editing. GK: Data curation, Investigation, Methodology, Writing – original draft. D-GL: Data curation, Investigation, Methodology, Writing – review & editing. YK: Data curation, Formal Analysis, Investigation, Supervision, Writing – review & editing. EC: Conceptualization, Data curation, Formal Analysis, Investigation, Methodology, Project administration, Supervision, Writing – review & editing. JL: Data curation, Formal Analysis, Investigation, Methodology, Supervision, Validation, Writing – review & editing. KW: Conceptualization, Data curation, Formal Analysis, Funding acquisition, Investigation, Methodology, Project administration, Supervision, Validation, Writing – original draft, Writing – review & editing.
