# Supplementary material for: Antimicrobial resistance genes harbored in invasive Acinetobacter calcoaceticus-baumannii complex isolated from Korean children during the pre-COVID-19 pandemic periods, 2015–2020
Source: Front Cell Infect Microbiol. 2024 Jul 4;14:1410997. doi: 10.3389/fcimb.2024.1410997 (PMC11254764; doi:10.3389/fcimb.2024.1410997)
Supplement: Supplementary file 1 [file DataSheet_1.docx]

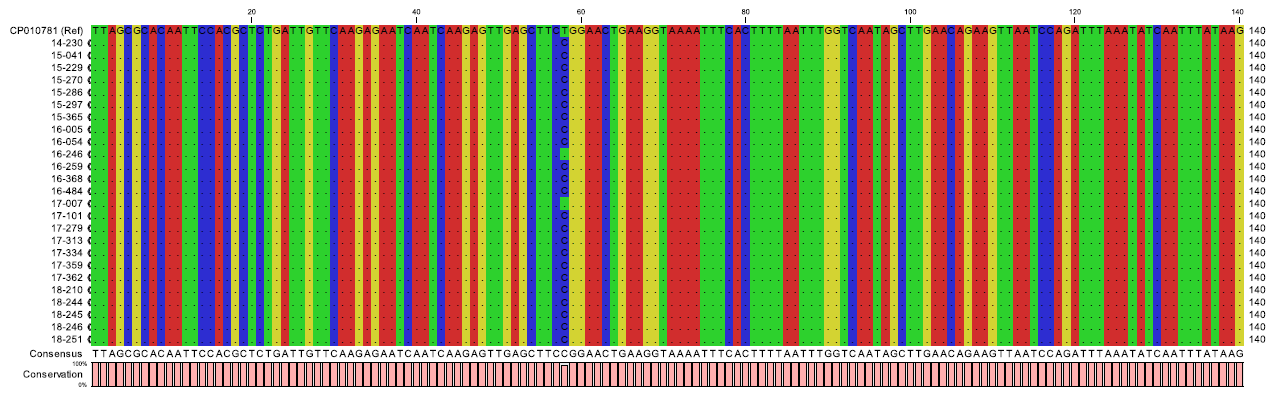

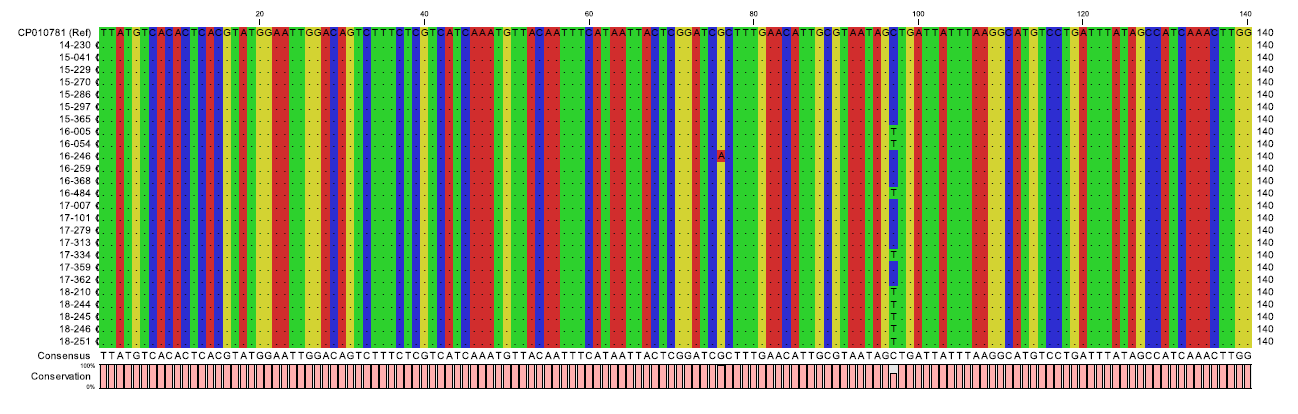


**Supplementary Figure 1. Comparative analysis of *lpx*A and *lpx*C genes with the reference *Acinetobacter baumannii* strain A1 sequence.** Several single nucleotide polymorphisms were observed.


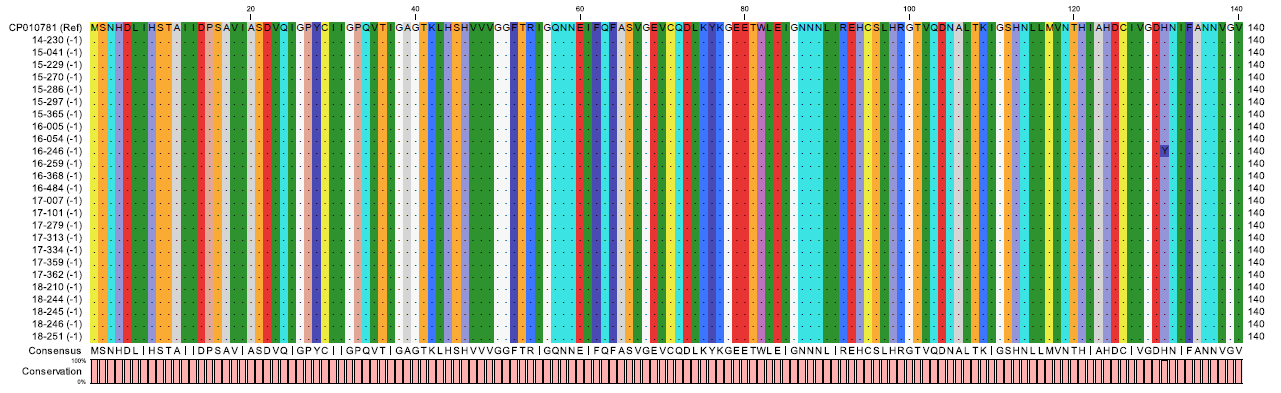

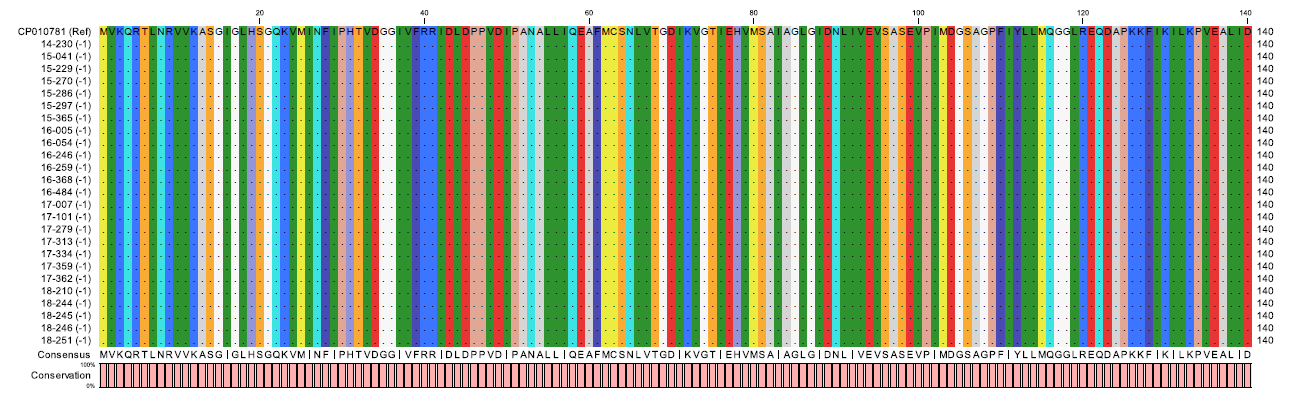


**Supplementary Figure 2. Comparative analysis of *lpx*A and *lpx*C amino acid sequence with the reference *Acinetobacter baumannii* strain A1 sequence.** Only one non-synonymous mutation was found from *lpx*A in one isolate, H135Y.
